# Editorial: Information extraction for health documents

**DOI:** 10.3389/frai.2023.1224529

**Published:** 2023-06-16

**Authors:** Enrico Mensa, Paloma Martínez Fernández, Roland Roller, Daniele P. Radicioni

**Affiliations:** ^1^Dipartimento di Informatica, Università degli Studi di Torino, Turin, Italy; ^2^Departamento de Informática, Computer Science and Engineering Department, Universidad Carlos III de Madrid, Leganés, Spain; ^3^German Research Center for Artificial Intelligence (DFKI), Berlin, Germany

**Keywords:** information extraction (IE), text mining (TM), electronic health records (EHR), concepts extraction, clinical narratives, unstructured medical data

Electronic health records (EHRs) gained a central role in many different medical and clinical settings: they typically contain salient information on patients in the form of unstructured, free-text data, such as clinical narratives, discharge summaries, outpatient care reports, prescription drug records, notes on past treatments by emergency departments, reports of laboratory studies, diagnostic texts associated with imaging, and more. All mentioned sources contain unstructured data that are valuable in decision-making processes and helpful in supplementing structured patient data: to figure out the relevance of unstructured data, we can consider that these account for 80% of the total of health-related data in a hospital (Kong, 2019). To make such information accessible, searchable, and available to different applications, the text needs to be transformed into structured data by many sorts of natural language processing (NLP), such as information extraction (IE) techniques and concept normalization to a structured ontology.

In the last few years a shift of computational paradigms from symbolic to distributed representations has deeply modified the landscape of the whole discipline of artificial intelligence, including NLP, and their specializations focused on medical data, as well. In short, this change in paradigms led to a dramatic increase in the level of performance ensured by systems relying on deep learning (DL) architectures: this fact is confirmed if one considers the architecture of the top-scoring systems involved in downstream tasks, such as GLUE and SUPERGLUE (Wang et al., 2018, 2019). DL systems are characterized by many technical changes; among the most prominent innovations, the costly feature selection of traditional Machine Learning systems has been superseded by automatic computing devices [such as the *attention* mechanism (Vaswani et al., 2017)], that are directly plugged into the system at the architectural level (Hahn and Oleynik, 2020). Also, typical DL architectures such as Transformers Networks (Devlin et al., 2018) are trained by employing huge amounts of text data in the order of hundreds of billions of tokens. Dealing with medical language requires models to deal with special linguistic phenomena, including telegraphic usages, abbreviations, acronyms, and syntactically ill-formed constructions (Mensa et al., 2020). Although some EHR data sets have been made available, such as i2b2 (Uzuner et al., 2011) and MIMIC (Johnson et al., 2016, 2018), these do not suffice to train models exclusively on medical data. To overcome these limitations, embeddings originally trained

on general language may be adapted to the medical language (Hahn and Oleynik, 2020). The medical setting is generally affected by the sparsity of corpora. There is broad consensus on the fact that insufficient clinical data sets are available in the public domain, primarily due to privacy issues and institutional concerns (Friedman et al., 2013), and that the inaccessibility of large-scale de-identified clinical corpora prevents clinical NLP from fully exploiting the architectures to date available.

Although various approaches have been proposed to cope with such limitations, e.g., based on transfer learning and domain adaptation to tailor pre-trained language models to the medical domain and on federated learning to deal with privacy issues (Cheng et al., 2020), many issues still require deepening research to provide equitable, secure, and ethical access to data. This Research Topic presents three works dealing with specific tasks, such as clinical concept recognition, the elaboration of clinical trials, and the automatic handling of numerical codes within EHRs.

Many approaches can be adopted to extract information from medical records. The contribution from Lossio-Ventura et al. focuses on *clinical concept recognition*, which constitutes the attempt to extract specific conceptual information from EHRs. The authors investigated six systems [CLAMP (Soysal et al., 2018), cTAKES (Savova et al., 2010), MetaMap (Jonquet et al., 2009), NCBO Annotator (Aronson and Lang, 2010), QuickUMLS (Soldaini and Goharian, 2016), and ScispaCy (Neumann et al., 2019)] against two benchmarks widely adopted in the field [i2b2 (Uzuner et al., 2011) and MIMIC-III (Johnson et al., 2016, 2018)]. The concept to be extracted are divided into four categories, namely: problem, treatment, test and anatomy. CLAMP achieved the best exact and inexact matching performance, with an *F*-score of 0.70 and 0.94, respectively. The authors also selected a subset of the MIMIC data to evaluate the systems on six characteristic challenges often found in medical texts. In particular, the six systems were executed on the sentences that contained negations, abbreviations, severity, ambiguity, and misspellings, showing that no single system excelled against all challenges. Instead, each system performed differently in particular tasks, showing that further work is required to develop algorithms that are resilient against this type of challenge.

The contribution from Luechtefeld et al. tackles instead a common issue concerning biomarker clinical trials publications: biomarker-based outcomes and eligibility criteria are very relevant in cancer clinical trials. However, most of the biomarker analyses reported for these trials are often difficult to access and with no controlled data access. Authors tackle this issue by proposing a new approach to semi-automating normalized, open-access data tables from published clinical trials of metastatic prostate cancer (mCRPC), employing a data curation and SER platform (Bozada et al., 2021). The authors extracted 585 hazard ratios, response rates, duration metrics, and 543 adverse events from 13 publications covering 10 clinical trials publication concerning mCRPC. The relevance of this data is shown by illustrating several use cases, such as the analyses of trial methods, comparison of treatment hazard ratios, and association of treatments with adverse events.

Finally, the paper from Deng and Denecke proposes a solution for common issues in medical procedures. Numerical codes often represent clinical procedures/injuries/body parts in medical documents to facilitate many processes, such as billing, quality assurance, and statistical analysis. These codes are often organized in taxonomies with hundreds or thousands of nodes, so the manual selection from human operators is intuitively unfeasible. Authors focus on using CHOP, the Swiss classification of surgical interventions system, which physicians use in daily practice to classify clinical procedures. The CHOP comprises more than 14,000 different classes at six levels. It aids operators in the code selection via a rule-based system composed of encoding experts and a manual search in the CHOP catalog. The authors investigate the possibility of automatic CHOP code generation based on a short query to enable automatic support of the manual classification. A thorough evaluation of many hierarchical classification systems shows that the per-node binary classification outperforms the non-terminal multi-class classification with an F1-micro measure between 92.6 and 94%.

## Author contributions

All authors equally participated in the concept of this Special Issue, and in writing the editorial.
